# Construction Strategies and Advances in Bone Marrow Microphysiological Systems

**DOI:** 10.3390/ijms27083586

**Published:** 2026-04-17

**Authors:** Tian Lin, Haodong Zhong, Qianyi Niu, Ruiqiu Zhang, Manman Zhao, Xiaobing Zhou

**Affiliations:** 1 National Center for Safety Evaluation of Drugs, National Institutes for Food and Drug Control, Beijing 102600, China; slint54@163.com (T.L.); 3323071873@stu.cpu.edu.cn (H.Z.); nosenosepro@163.com (Q.N.); ruiqiuzhang0925@163.com (R.Z.); 2 State Key Laboratory of Natural Medicines, China Pharmaceutical University, Nanjing 211198, China; 3 School of Pharmacy, Shenyang Pharmaceutical University, Shenyang 110016, China; 4 National Institutes for Food and Drug Control, Chinese Academy of Medical Sciences & Peking Union Medical College, Beijing 100730, China

**Keywords:** bone marrow, microphysiological system, organ-on-a-chip, organoid, hematopoietic stem cell

## Abstract

Bone marrow(BM) is the primary site of hematopoiesis, supporting the self-renewal and differentiation of hematopoietic stem cells (HSCs). Its function depends on a highly complex microenvironment composed of stromal cells, vascular networks, extracellular matrix components, and dynamic biophysical signals. Traditional two-dimensional culture systems and animal models fail to adequately recapitulate the spatial architecture and dynamic regulatory processes of the human bone marrow niche, thereby limiting in-depth investigations into hematopoietic regulatory mechanisms, disease pathogenesis, and drug-induced bone marrow toxicity. In recent years, advances in microphysiological systems (MPS) have provided novel engineering approaches for the in vitro reconstruction of the bone marrow microenvironment. This review systematically summarizes current construction strategies for bone marrow MPS, including three-dimensional self-organized bone marrow organoids and microfluidic bone marrow-on-a-chip platforms. Particular attention is given to the roles of key cellular components, biomaterial scaffolds, vascularized architectures, and dynamic perfusion systems in biomimetic bone marrow engineering. In addition, we discuss strategies for constructing more complex models, such as vascular niches, vascularized bone tissue constructs, and bone metastasis models. Bone marrow MPS more faithfully recapitulate the hematopoietic microenvironment and provide a physiologically relevant in vitro platform for hematopoietic research, disease modeling, and drug evaluation, thereby supporting future advances in precision and regenerative medicine.

## 1. Introduction

Bone marrow (BM) is one of the primary hematopoietic organs in the human body, located within the medullary cavities of long bones and flat bones. It is responsible for the maintenance, self-renewal, and multipotent differentiation of hematopoietic stem cells (HSCs) [1]. Unlike other solid organs, BM exhibits a highly complex three-dimensional architecture and a dynamic microenvironment. Its functional maintenance relies on the intricate regulation among diverse cell types, extracellular matrix components, and soluble factors [2]. The fate of HSCs is not determined by a single signal but is shaped by the entire hematopoietic microenvironment. This microenvironment includes osteoblasts, osteoclasts, mesenchymal stem cells (MSCs), endothelial cells, macrophages, and neuromodulatory components, collectively forming a biological system with a highly integrated structure and function [3].

In past BM research and BM toxicity evaluation systems, animal models and two-dimensional cell culture have been the most commonly used tools, but both possess specific limitations. Traditional in vitro two-dimensional culture systems can only provide planar growth conditions, making it difficult to authentically reproduce the spatial structure and mechanical properties of the BM microenvironment. This leads to rapid differentiation or loss of pluripotency of HSPCs during in vitro culture, rendering this system unsuitable for recapitulating physiological hematopoietic regulation [4]. While animal models can partially mimic in vivo environments, species differences, ethical constraints, and limited experimental controllability limit their utility in precise mechanistic studies and personalized drug evaluation [5]. Particularly in research on leukemia pathogenesis, BM failure syndrome, and tumor bone metastasis, traditional models often fail to accurately reflect the true state of the human BM microenvironment [6].

Against this backdrop, the rapid advancement of microphysiological systems technology (MPS) has opened new technical avenues for in vitro reconstruction of BM. MPS utilize micro-scale cell culture platforms to simulate the functional characteristics of specific tissues or organs by exposing cells to microenvironments that mimic their functional or pathophysiological conditions, thereby modeling their functional features in vitro [7]. For bone marrow tissue modeling, multiple in vitro strategies have been developed, including scaffold-based 3D cultures, organoids, and microfluidic systems. Among these, two major approaches have attracted particular attention: one relies on bone marrow organoids (BMOs) constructed based on cellular self-organization capabilities, while the other utilizes microfluidic chip technology to create BM-on-a-chip systems [8]. The former emphasizes the spontaneous biological self-organization and natural reconstruction of cell–cell interactions, while the latter prioritizes structural controllability, hydrodynamic regulation, and long-term dynamic culture capabilities. Both approaches strive to enhance the physiological relevance and functional stability of BM models [9].

Through the integration of materials science, microfluidic engineering, and bioprinting technologies, BM MPS have progressed from conceptual development to functional validation and application expansion. Currently, these models are widely applied in hematopoietic stem cell maintenance and expansion studies, hematological disease modeling, drug toxicity assessment, and precision medicine exploration, demonstrating clear advantages over traditional two-dimensional culture systems [10].

Accordingly, this review systematically summarizes the construction strategies and technological advances of BM MPS, focusing on elucidating the design logic and functional characteristics of various in vitro models. It summarizes their specific applications in hematopoietic regulation, disease modeling, and drug evaluation. Concurrently, it analyzes key challenges and future development directions in this field based on the current research status, aiming to provide systematic references for optimizing in vitro biomimetic models of BM.

## 2. Construction of BM MPS

**Figure 1:**
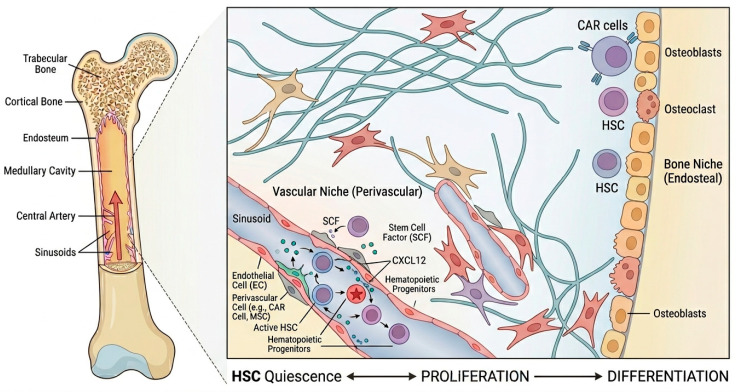
Schematic illustration of the structural organization and regulatory mechanisms of the bone marrow microenvironment. The endosteal and perivascular niches, together with the mesenchymal stromal network, coordinately regulate hematopoietic stem and progenitor cell function through cell–cell interactions, signaling gradients, and mechanical cues. Arrows indicate regulatory interactions between niche components.

The in vitro reconstruction of the BM microenvironment fundamentally involves the engineered reproduction of its spatial structure and function. Natural BM comprises structural units formed by the endosteal niche, perivascular niche, and mesenchymal support network, with hematopoietic homeostasis dependent on the coordinated regulation of cell interactions, signaling gradients, and mechanical microenvironments [6,11]. A schematic representation of the bone marrow microenvironment is provided in Figure 1 to illustrate its spatial organization and regulatory complexity.

Therefore, constructing a BM MPS requires balancing structural biomimicry with functional controllability, rather than merely replicating three-dimensional culture conditions. Based on these biological characteristics, current BM MPS can be broadly categorized into two representative strategies. One approach employs three-dimensional biomaterial matrices to support cellular self-organization and reconstruct the spatial architecture and hierarchical organization of the BM, typically in the form of organoids. The other utilizes microfluidic platforms to enable precise control of vascular niches, cell migration, and molecular transport through structural compartmentalization and dynamic perfusion. Although these strategies differ in design principles and scales, both aim to recapitulate the functional complexity of the BM niche in vitro [12,13].

### 2.1. Biological Basis of the Bone Marrow Microenvironment

The BM serves as the primary hematopoietic organ in the human body, with its function dependent on a highly organized and dynamically regulated microenvironmental system. Hematopoietic stem and progenitor cells (HSPCs) are not randomly distributed throughout the BM cavity but instead reside within specific stem cell niches. This concept was first proposed by Schofield in 1978 to describe the localized tissue environment that maintains stem cells in an undifferentiated state [14].

From a spatial anatomical perspective, the BM microenvironment is not a single entity but comprises multiple functionally overlapping niches. The classical view divides it into the endosteal niche and the perivascular niche. The endosteal region, adjacent to the trabecular bone surface, allows HSPCs to directly interact with osteoblasts and related bone matrix cells. This area is believed to contribute to maintaining the quiescent state and long-term reconstitution capacity of HSPCs [2]. In the perivascular niche, endothelial cells and surrounding mesenchymal stem cells form a critical supportive structure. They maintain HSC retention and functional homeostasis by secreting molecules such as stem cell factor (SCF) and CXC chemokine ligand 12 (CXCL12) [15]. At the cellular composition level, the BM microenvironment exhibits high heterogeneity. Perivascular mesenchymal stromal cells form the core components of the HSC niche, including subpopulations such as Leptin receptor (LepR)-expressing stromal cells, Nestin-GFP-labeled stromal cells, and CXCL12-abundant reticular (CAR) cells. Endothelial cells not only constitute vascular structures but also participate in regulating HSPC fate by expressing Notch ligands and other signaling molecules [16]. Macrophages and sympathetic-associated cells exert indirect regulatory effects primarily by modulating niche structural stability and HSPC mobilization processes [17]. The physical and metabolic characteristics of the BM microenvironment also decisively influence HSPC function. A pronounced oxygen gradient exists within the marrow, with long-term HSPCs typically residing in relatively hypoxic regions. They maintain their stemness through the hypoxia-inducible factor (HIF) signaling pathway and glycolytic metabolism [18]. Concurrently, mechanical properties of the BM matrix—such as elasticity and stiffness—have been demonstrated to influence HSPC fate determination. Studies indicate that lower-stiffness environments favor maintaining HSPC undifferentiated states, whereas high-stiffness conditions tend to promote differentiation [19].

**Figure 2:**
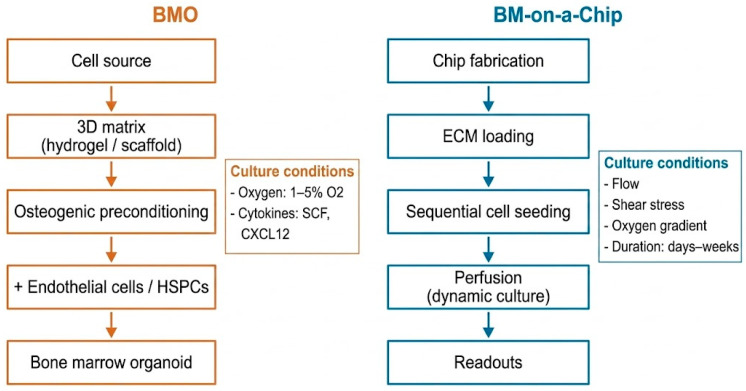
Representative construction protocols for BMO and BM-on-a-chip systems. BMOs rely on 3D matrix-supported self-organization, whereas BM chips are based on microfluidic engineering and dynamic perfusion. Key culture conditions, including oxygen, cytokines, flow, and culture duration, regulate system functionality.

Collectively, the BM microenvironment constitutes a dynamic system shaped by multicellular interactions, molecular signaling networks, mechanical properties, and metabolic regulation. Its complexity renders traditional two-dimensional culture systems inadequate for comprehensive simulation, driving the development of MPS like BMOs and BM-on-a-chip. To illustrate the engineering strategies for reconstructing the bone marrow microenvironment, representative construction workflows and key culture conditions of BM organoids and BM-on-a-chip systems are summarized in Figure 2.

### 2.2. Strategies for BMO Construction

BMOs refer to miniature tissue structures that reconstruct the characteristics of BM tissue in vitro through three-dimensional culture systems. These structures contain key elements of the hematopoietic niche, supporting active endogenous hematopoiesis while promoting the growth and survival of hematopoietic cells from adult donors [20]. Their core objective lies in mimicking the spatial organization and functional interactions between HSCs and BM stromal cells.

The primary step in BMO construction is establishing an appropriate cell source. Research generally recognizes MSCs as the core cells for organoid formation. They not only provide structural support for the three-dimensional architecture but also secrete key regulatory factors such as SCF and the chemokine CXCL12, maintaining the quiescent state and long-term function of HSCs [15]. Studies indicate that culturing MSCs seeded onto three-dimensional scaffold materials induces the formation of a reticular structure resembling BM stroma, establishing a microenvironment that supports hematopoietic cell adhesion and survival. Bourgine et al. [21] successfully induced human MSCs to form bone-like endosteal structures by culturing them on a three-dimensional scaffold composed of collagen and hydroxyapatite. This scaffold further supported the long-term maintenance and expansion of HSPCs, demonstrating that MSCs can spontaneously form functional hematopoietic support microenvironments in vitro.

Natural BM is a highly vascularized tissue, accounting for 10–15% of total cardiac output [22]. The vascular system not only facilitates material exchange but also regulates hematopoietic stem cell homeostasis and differentiation by secreting multiple factors [23]. Therefore, incorporating endothelial cells into organoid construction significantly enhances the physiological relevance of the model. Previous studies have established a three-dimensional co-culture system by mixing human bone marrow-derived mesenchymal stem cells (hBM-MSCs), osteogenically differentiated hBM-MSCs, and human umbilical vein endothelial cells (HUVECs) in specific ratios and embedding them in fibrin gel. This system formed a functional microvascular network within 4 days of culture. Endothelial cells expressed vascular endothelial cadherin (VE-cadherin) and tight junction protein ZO-1, indicating the formation of a barrier-like vascular wall structure [21]. These findings demonstrate that the synergistic self-organization of MSCs and endothelial cells can reconstruct a vascularized BM microenvironment with structural and functional characteristics in vitro. This provides an experimental platform closer to in vivo conditions for simulating the perivascular niche and studying HSC–matrix interactions [24].

Induced pluripotent stem cells (iPSCs) offer a novel cellular source for constructing BMOs. Frenz-Wiessner et al. [25] utilized human iPSCs, inducing them through staged differentiation into mesodermal, endothelial precursor, and mesenchymal-like cells, then promoting their self-organization within a three-dimensional extracellular matrix (ECM) scaffold. The resulting endothelial-like luminal structures exhibited spatial organization resembling the spatial organization of BM sinusoids, regulating hematopoietic stem/progenitor cell homing and migration while enabling long-term in vitro production of mature lineages including granulocytes and monocytes. This approach offers the advantage of deriving diverse microenvironmental cells from a single pluripotent stem cell line, enhancing the consistency of tissue composition.

Biomaterials for BMO construction must simultaneously provide structural support, biological activity, and controllability. Their primary function is to supply a three-dimensional spatial framework while mimicking the mechanical properties and extracellular matrix environment of BM. Natural materials such as collagen and Matrigel can exhibit excellent biocompatibility and cell adhesion properties, promoting cellular self-organization into BM-like structures [26]. Ventura et al. [27] performed heterotopic transplantation in mice by co-implanting human MSCs and HSPCs into degradable scaffolds coated with Matrigel or collagen I/III gel. Results demonstrated that this engineered microenvironment formed marrow-like tissue structures within the host, supporting the survival and differentiation of human hematopoietic cells, and partially restoring functional hematopoietic activity at the heterotopic site [27]. On the other hand, mineralized materials such as hydroxyapatite can mimic the stiffness and mineral composition of bone tissue, thereby inducing MSCs toward osteogenic differentiation and establishing a microenvironment closer to the endosteal niche [28]. Furthermore, synthetic materials like polyethylene glycol (PEG) and poly(lactic-co-glycolic acid) (PLGA) enable precise regulation of cellular behavior by modulating stiffness, pore size, and surface ligands, providing crucial tools for constructing standardized BMOs [29,30]. To further summarize the representative biomaterials currently employed in BMO construction and their functional roles in reconstructing the bone marrow microenvironment, a comparative overview is provided in Table 1. This table highlights commonly used natural, mineralized, and synthetic biomaterials, along with their key physicochemical properties and specific contributions to organoid formation, including structural support, regulation of cell behavior, and niche-specific functionality.

In summary, the construction of BMOs relies on the synergistic interaction of stromal cells, vascular cells, hematopoietic cells, and biomaterial scaffolds. Through self-assembly or engineered approaches, these components form three-dimensional structures exhibiting the functional characteristics of BM. Such models emphasize the reconstruction of microenvironmental architecture and the restoration of intercellular interaction networks, thereby reproducing key components of the BM niche in vitro. In addition to structural design, BMO functionality critically depends on defined culture conditions, including oxygen tension, cytokine supplementation, and matrix mechanical properties, which collectively regulate stem cell behavior and niche formation.

### 2.3. Strategies for Bone Marrow Chip Construction

Organ-on-a-chip (OoC) utilizes microfabrication techniques to create microfluidic chips, co-culturing multiple human cell types within microchannels according to physiological architecture to replicate the fundamental structure and function of human organs. Microfluidic technology operates at the micro- and nanoscale and enables precise control of fluid flow within engineered microchannels. Its core component, the microfluidic chip, employs precisely engineered microchannels to control fluid flow, enabling dynamic regulation of the cellular microenvironment [38]. Unlike static culture systems, microfluidic platforms can mimic the human vascular system by continuously delivering nutrients and metabolites, generating stable fluid shear forces and concentration gradients. This enables better maintenance of cellular function and differentiation potential [39,40].

Based on research applications, existing BM chips can be further categorized into normal hematopoietic BM chips, vascular niche BM chips, bone tissue–BM coupled chips, tumor BM microenvironment chips, and BM toxicity/disease model chips. This functional classification facilitates systematic summarization of each model’s application value and developmental direction. To provide a structured overview of current BM-on-a-chip technologies, representative platforms and their key characteristics are summarized in Table 2. This comparison highlights the integration of cellular components, microenvironmental features, and functional applications, illustrating how distinct chip designs enable the reconstruction of specific bone marrow niches for diverse experimental objectives. Compared with organoid systems, BM-on-a-chip platforms enable more precise control of dynamic culture conditions, such as flow rate, shear stress, and oxygen gradients, which are essential for reproducing vascular and hematopoietic microenvironments.

#### 2.3.1. Hematopoietic BM Chips

Simulating in vivo hematopoiesis and sustaining HSPC function represent the most fundamental application goals of BM chips. Long-term maintenance of HSPCs in vivo relies on physical support and molecular signaling regulation provided by the BM niche [48]. However, under conventional two-dimensional culture conditions, HSPCs typically undergo differentiation within a short period and lose their self-renewal capacity, severely limiting their in vitro research and clinical applications. Therefore, researchers have constructed three-dimensional structures within microfluidic chips and introduced marrow-associated cells to mimic the marrow niche and sustain hematopoietic function. The development of BM chips typically involves multiple aspects, including the integration of key cellular components, microfluidic chip structural design, matrix material construction, and microenvironment parameter regulation.

HSPCs represent one of the most critical functional cell types in BM chip construction, typically derived from human umbilical cord blood or BM aspiration samples. The most commonly used surface markers for HSPCs in clinical and research settings are CD34^+^CD38^−^, where CD34 serves as a key surface glycoprotein marker for hematopoietic stem cells and early progenitor cells, while CD38 negativity is typically associated with a highly undifferentiated state and multipotency [49]. HSPCs from different sources may exhibit variations in proliferation capacity, in vitro survival rates, and differentiation potential. For instance, umbilical cord blood-derived HSPCs typically demonstrate higher proliferative activity and lower immunogenicity, making them particularly valued in regenerative medicine and in vitro toxicity studies [50]. MSCs possess multipotent differentiation potential toward osteogenic, chondrogenic, adipogenic, and other lineages. Within the BM stroma, they primarily serve a supportive role, regulating HSPC adhesion, homing, proliferation, and differentiation through the secretion of multiple cytokines [51]. This provides HSPCs with in vivo-like physical and biochemical support, enhancing the physiological relevance of chip systems. Torisawa et al. [28] pioneered the implantation of mouse bone tissue into a microfluidic device, successfully constructing a functional BM chip. They demonstrated that this system maintains hematopoietic stem and progenitor cell populations while continuously generating myeloid and lymphoid cells, indicating the BM chip’s ability to recreate the in vitro hematopoietic microenvironment. Sieber et al. [41] utilized a three-dimensional porous ceramic scaffold combined with MSCs to construct a BM MPS, achieving long-term culture of human HSPCs for over four weeks and demonstrating that this system can maintain hematopoietic function.

In terms of chip material and structural design, microfluidic chip fabrication typically employs materials such as polydimethylsiloxane (PDMS) that exhibit excellent biocompatibility, gas permeability, and optical transparency. These materials not only facilitate the construction of micrometer-scale channels and independent chambers but also accommodate diverse experimental requirements [52]. Torisawa et al. [28] abricated a central cylindrical cavity with one open end using PDMS. After filling the cavity with type I collagen gel and implanting it subcutaneously in mice, the engineered BM formed a cylindrical structure comprising both cortical and cancellous bone containing hematopoietic cells. Its hematopoietic cell composition resembled that of natural BM. After removing the chip from the mouse, perfusion culture in a microfluidic device not only improved cell survival rates but also reduced interference from metabolic waste accumulation on experimental results, providing a novel approach for studying BM drug responses and hematological diseases. The BM chip model developed by Kefallinou et al. [53] comprises interconnected culture chambers and perfusion channels. It enables three-dimensional co-culture of MSCs and HSPCs within a sealed PDMS-based microfluidic device, thereby reconstructing and sustaining the hematopoietic microenvironment in vitro. MSCs first adhere within the chip and form a supportive matrix-like microenvironment, followed by the introduction of HSPCs. This allows HSPCs to interact directly with MSCs without requiring exogenous scaffolds. This construction emphasizes simulating the in vivo hematopoietic niche through spatial confinement, dynamic fluid environments, and cell–cell contacts, thereby achieving long-term survival, phenotypic maintenance, and functional stability of hematopoietic stem/progenitor cells.

The dynamic perfusion system is one of the key features distinguishing the BM chip from traditional models. Micro-pumps or gravity-driven devices enable continuous fluid circulation within the chip, mimicking BM blood flow while maintaining stable nutrient supply and metabolic waste clearance [54]. Additionally, the microfluidic system establishes oxygen concentration gradients, simulating the hypoxic microenvironment present in BM, which is critical for maintaining HSC quiescence [16].

Through the integration of microfluidic structures, biomaterial scaffolds, key cellular components, and dynamic perfusion systems, BM chips achieve highly realistic simulation of the BM microenvironment’s structure and function. Compared to traditional culture systems, BM chips enable more precise control of microenvironmental parameters and sustain long-term hematopoiesis. This provides a vital technological platform for studying hematopoietic regulatory mechanisms, disease modeling, and drug screening, representing a key direction in the development of BM MPS.

#### 2.3.2. Vascular Niche BM Chips

Within the BM microenvironment, the vascular system not only transports blood cells but also constitutes a critical niche structure regulating HSPC fate. In vivo studies indicate that most HSPCs localize to BM sinusoids and periarterial regions, forming tightly integrated functional units with vascular endothelial cells and surrounding MSCs—the vascular niche. Vascular endothelial cells maintain HSPC quiescence and self-renewal capacity by secreting signaling molecules such as SCF, CXCL12, and Notch ligands. Concurrently, blood flow shear stress and vascular permeability regulate hematopoietic cell migration and mobilization processes [55]. For in vitro BMOs and BM chip platforms, the absence of mature vascular structures constitutes a bottleneck limiting long-term hematopoietic maintenance and immune cell maturation. In traditional spheroid organoids, limited endogenous angiogenesis efficiency often leads to central hypoxia, metabolic waste accumulation, and restricted cell migration pathways, limiting their ability to reproduce the physiological gradients of the hematopoietic niche [55]. Therefore, reconstructing structurally intact and functionally active vascular networks within BM chips is a crucial strategy for achieving physiological BM microenvironment mimicry.

Vascular niche BM chips typically utilize microfluidic devices in which perfusable three-dimensional matrix chambers are constructed to co-culture endothelial cells, MSCs, and HSPCs, thereby inducing endothelial cells to form microvascular networks. Commonly used matrix materials include type I collagen, fibrin, and gelatin methacrylate (GelMA) hydrogels, which provide a suitable mechanical environment for vascular formation while supporting cell migration and lumen formation.

Nelson et al. [42] reported a multi-niche microvascularized human BM chip integrating three functional zones within the device: an endosteal niche, a central marrow space, and a perivascular niche. The base of the chip utilized hBM-MSCs induced toward osteogenic differentiation to form a mineralized matrix layer, simulating the endosteal microenvironment. This demonstrated that the vascular niche significantly enhanced HSC survival and maintained CD34 expression. Endothelial cells and MSCs were seeded within an upper fibrin network to form a three-dimensional microvascular network; the central region served for CD34^+^ HSPC culture. This spatially separated yet functionally interconnected design enables independent analysis of how different niches regulate hematopoietic function.

Overall, the vascular niche BM chip successfully reconstructs the vascular niche regulating HSPC function by integrating a three-dimensional matrix, a self-assembled endothelial vascular network, and a dynamic perfusion system. This model emphasizes the simulation of vascular structure and blood flow environment regulation on hematopoiesis and cell migration, providing a crucial in vitro platform for studying hematopoietic stem cell biology, drug delivery, and BM toxicity.

#### 2.3.3. Vascularized Bone Tissue Chips

In natural BM, bone tissue and the marrow cavity do not exist independently but form an integrated bone and marrow system through complex structural and functional interactions. The endosteal niche formed on the surface of mineralized trabeculae is considered a critical region for maintaining HSC quiescence and long-term self-renewal. Osteoblasts within bone tissue participate in hematopoietic regulation by secreting SCF, osteopontin (OPN), and other regulatory factors [56]. While traditional vascular niche BM chips can simulate vascular-BM interactions, they lack mineralized bone structures and cannot reproduce the endosteal niche. Consequently, recent studies have introduced mineralized bone tissue, BM matrix, and functional vascular networks into microfluidic chips to achieve higher-level reconstruction of the BM microenvironment [57].

The construction strategy for such chips typically relies on microfluidic platforms. A porous scaffold composed of collagen, fibrin, or hydroxyapatite is introduced into the central culture chamber of the chip [47]. MSCs or osteoprogenitor cells are then seeded onto this scaffold and cultured under osteogenic induction conditions for several weeks. This process induces the cells to secrete a collagen matrix and form mineralized bone tissue, establishing a three-dimensional structure with trabecular features [56]. Following bone tissue formation, endothelial cells are introduced to induce vascular network development, thereby constructing bone tissue structures with perfusable microvascular systems. This strategy simultaneously mimics bone matrix mineralization and intrabone vascular architecture, achieving in vitro reconstruction of the bone-vascular microenvironment.

Jusoh et al. [43] developed a microfluidic vascularized bone tissue model integrating a three-dimensional microvascular network with a biomimetic bone tissue microenvironment on a single chip, enabling in vitro reconstruction of bone-vascular interactions. The device comprises multiple parallel microchannels with distinct functional compartments dedicated to angiogenesis, bone tissue construction, and nutrient exchange. The study employed fibrin infused with hydroxyapatite (HA) nanoparticles as the extracellular matrix to mimic the highly porous, interconnected mineralization characteristics of natural bone tissue. Simultaneously, fibroblasts were introduced as paracrine support cells, providing proangiogenic factors and matrix proteins to promote luminal formation and networked growth of endothelial cells within the three-dimensional environment. Results indicate that modulating HA content in the matrix significantly influences vascular network formation, suggesting that matrix mineralization levels play a critical role in regulating bone tissue vascularization.

Bersini et al. [58] established a three-dimensional co-culture system. By co-culturing endothelial cells and MSCs in a biomimetic matrix, they constructed a vascularized three-dimensional model exhibiting bone tissue characteristics. This study systematically evaluated the effects of multiple key parameters—including oxygen tension, cell seeding density and ratio, medium composition, and hydrogel type—on vascular network formation and bone-like tissue development. By analyzing the correlations among different regulatory factors, precise control over both angiogenesis and osteogenic differentiation processes was achieved. This model validated the central role of interactions between endothelial cells and osteogenic cells in constructing vascularized bone-like tissue. It further demonstrated that the mechanical properties and composition of the matrix can simultaneously influence vascular network maturation and osteogenic differentiation, providing a systematic approach for establishing controllable and reproducible vascularized bone tissue models in vitro.

Vascularized bone tissue chips more closely resemble the structural and functional characteristics of the actual BM microenvironment, representing one of the most structurally complex models within current BM MPS [23]. In the future, with advancements in 3D bioprinting and microfluidic technologies, such systems are expected to achieve precise reconstruction of trabecular bone structures and dynamic blood flow regulation. This will provide a more reliable platform for studying hematopoietic regulatory mechanisms, modeling bone metastatic tumors, and conducting drug screening.

#### 2.3.4. Cancer Bone Metastasis Chips

Bone serves as a critical metastatic target organ for various malignancies. Cancer cells migrate to the bone microenvironment via the circulatory system, colonizing and surviving within BM. Their homing and metastatic capabilities are jointly regulated by intrinsic tumor cell characteristics and bone microenvironmental signals [59]. Within bone tissue, metastatic cancer cells can remain dormant for extended periods until activated by local or systemic stimuli, triggering bone metastasis [60]. Bone metastasis not only causes clinical complications such as bone pain, pathological fractures, spinal cord compression, and hypercalcemia but also significantly shortens patient survival [61]. Traditional in vivo and in vitro models struggle to accurately recreate the interactions among tumor cells, osteocytes, and vascular endothelial cells during bone metastasis. Advances in microfluidic technology have enabled the reconstruction of the bone tissue microenvironment in vitro, providing a new platform for studying the mechanisms of tumor bone metastasis and drug interventions.

Bersini et al. [62] developed a three-dimensional microfluidic model specifically designed to study breast cancer bone metastasis. In this system, hBM-MSCs were first seeded into the matrix compartment of the chip and cultured under osteogenic induction conditions for approximately 2–3 weeks, enabling the deposition of bone-like extracellular matrix within a three-dimensional collagen matrix. Subsequently, endothelial cells were seeded into adjacent perfusion channels to form a monolayer endothelial barrier covering the channel surface, simulating the structure of the vascular endothelium. Upon model maturation, breast cancer cells were introduced into the vascular channels to monitor their migration through the endothelial layer, invasion into the bone-like matrix, and formation of micrometastatic foci. This construction method clearly separates the vascular and bone matrix functional zones, providing a highly controllable experimental platform for analyzing tumor bone metastasis processes.

Hao et al. [63] developed a three-dimensional bone tissue chip model based on a synchronous growth dialysis mechanism to simulate breast cancer bone metastasis. This strategy utilizes a dialysis membrane for continuous exchange of low-molecular-weight nutrients and metabolic waste while promoting gradual accumulation of macromolecular proteins within the cell culture compartment, thereby supporting long-term maturation of bone-like tissue. The chip structure is divided by a dialysis membrane into an upper culture medium channel and a lower cell growth chamber. MC3T3-E1 osteoprogenitor cells were seeded onto a surface-etched glass substrate. After continuous culture for 30 days, mature three-dimensional bone tissue approximately 85 μm thick and rich in mineralized collagen fibers formed within the chip. This bone metastasis chip offers miniaturization and high-throughput advantages, enhancing interactions between cancer cells and the bone matrix.

Marturano-Kruik et al. [44] established a vascularized bone niche model by co-culturing hBM-MSCs and endothelial cells within a collagen matrix, inducing the formation of a perfusable microvascular network with luminal structures in the central compartment. This approach enables the integration of vascular and bone tissue components within a single platform, thereby reconstructing a bone–vascular niche that closely resembles in vivo conditions. Such systems provide a physiologically relevant microenvironment for studying tumor cell extravasation, early metastatic colonization, and implantation behavior within the bone marrow.

## 3. Applications of BM MPS

### 3.1. Research on Normal Hematopoietic Processes and Hematopoietic Niche Mechanisms

The most fundamental and core application of BM MPS is to reconstruct the in vitro hematopoietic process and elucidate the interactions between HSPCs and their microenvironment. Traditional two-dimensional culture systems struggle to maintain long-term HSPC pluripotency and cannot replicate the complex cell–matrix and vascular regulatory mechanisms within the BM niche. In contrast, BM MPS effectively address these limitations through three-dimensional architecture and multicellular co-culture [64]. The three-dimensional architecture, stiffness, and cellular configuration of organoids more closely resemble those of native BM, enabling researchers to analyze paracrine signaling and contact-dependent interactions between HSPCs and their surrounding matrix in vitro [45]. The BM chip developed by Torisawa et al. successfully maintained normal HSPC proportions and supported multiple hematopoietic cell lineages by implanting engineered bone tissue into a microfluidic device for continuous perfusion culture, demonstrating the system’s ability to reconstruct functional hematopoietic microenvironments in vitro [28].

Furthermore, BM chips can investigate niche-specific regulation of HSPC fate. For instance, constructing distinct vascular endothelium and stromal layers within the chip simulates the perivascular niche, enabling observation of HSPC migration, adhesion, and differentiation [57]. Thus, BM MPS provide a research platform closer to in vivo conditions than traditional in vitro cultures for deciphering hematopoietic regulatory mechanisms. BM MPS can also investigate hematopoietic stem cell migration and homing mechanisms. Studies demonstrate that within BM chips containing vascular structures, HSPCs migrate along CXCL12 concentration gradients and localize to perivascular regions—a phenomenon consistent with in vivo observations [3]. Furthermore, by modulating fluid shear stress, the system can simulate HSC entry into and exit from the BM, providing a crucial tool for studying hematopoietic stem cell mobilization mechanisms. Consequently, the BM MPS not only supports long-term hematopoiesis but also offers a vital in vitro model for elucidating the regulatory mechanisms governing the hematopoietic niche.

### 3.2. Mechanistic Insights Enabled by BM MPS

Beyond recapitulating hematopoietic processes, BM MPS provide a unique experimental platform for dissecting fundamental biological mechanisms that are otherwise difficult to isolate in vivo. By enabling independent control over cellular composition, biochemical gradients, and physical parameters, these systems allow researchers to move from descriptive observation toward causal analysis.

One key advantage of BM-on-a-chip systems lies in their ability to establish controlled chemokine gradients. This enables precise investigation of how the CXCL12–CXCR4 signaling axis governs HSPC homing and retention within specific niches, and helps distinguish gradient-driven migration from adhesion-mediated localization mechanisms [15]. Additional studies further demonstrate that CXCL12 gradients regulate cytoskeletal polarization and directional migration of HSPCs, highlighting its central role in niche localization [65].

In addition to biochemical regulation, BM MPS allow systematic dissection of niche-derived cellular interactions. By selectively incorporating endothelial and stromal components, these models enable mechanistic analysis of how vascular niche signals regulate HSPC maintenance, quiescence, and lineage commitment [22]. Emerging evidence also indicates that endothelial–stromal crosstalk and niche remodeling critically influence HSPC function and disease progression [66].

Moreover, BM MPS provide an effective platform to investigate the role of physical microenvironmental cues in hematopoiesis. Through the use of tunable biomaterials, matrix stiffness can be precisely modulated, allowing direct evaluation of how mechanical properties influence stem cell fate decisions [67].

Microfluidic systems further extend this capability by enabling dynamic control of flow and shear stress. This allows mechanistic exploration of how vascular perfusion influences cell behavior, nutrient transport, and niche stability [39]. In vivo imaging studies further confirm that HSPC homing involves flow-dependent rolling, adhesion, and transendothelial migration within bone marrow microvessels [68].

Collectively, BM MPS transform in vitro models from passive representations of tissue structure into active tools for mechanistic discovery, enabling systematic interrogation of biochemical, cellular, and physical regulation within the bone marrow microenvironment.

### 3.3. Drug Toxicity Evaluation and Drug Screening

The BM serves as a target organ for toxicity in various antitumor therapies, with chemotherapy drugs, molecularly targeted agents, and radiotherapy all capable of inducing varying degrees of BM suppression [68]. However, traditional animal models are limited by significant species differences, often failing to accurately predict hematopoietic toxicity in humans. Marrow MPS constructed from human cells can recreate the hematopoietic microenvironment in vitro, thereby enhancing the physiological relevance and predictive capability of drug-induced BM toxicity assessments. The human BM chip developed by Chou et al. successfully reproduced multiple clinically induced myelotoxicity responses. Administration of the Aurora B kinase inhibitor barasertib significantly reduced neutrophil production while relatively preserving erythroid cells, consistent with clinical observations. Furthermore, hematopoietic function gradually recovered after drug withdrawal, demonstrating the system’s ability to simulate both BM injury and recovery processes [46]. The same study also revealed that the anticancer drug 5-fluorouracil significantly reduced hematopoietic progenitor cell numbers, while MSCs and endothelial cells exhibited relative tolerance, indicating differential drug sensitivity among BM cell types [46].

The BM chip has also been employed to study radiation-induced BM injury. Torisawa et al. observed a significant reduction in hematopoietic cells following ionizing radiation exposure in the BM chip and successfully promoted hematopoietic recovery using granulocyte colony-stimulating factor (G-CSF), demonstrating the chip’s ability to simulate radiation therapy damage and therapeutic responses [28]. Furthermore, the BM MPS facilitates drug screening and dose optimization. Its real-time monitoring of hematopoietic cell counts and lineage ratios enables assessment of drug effects across concentrations to determine safe dosage ranges [69]. Compared to conventional cell cultures, this system simultaneously evaluates multiple cell types, enhancing toxicity prediction accuracy.

Thus, the BM MPS provides a highly physiologically relevant human platform for drug toxicity evaluation and drug screening, offering the potential to increase drug development success rates and reduce animal testing.

### 3.4. Construction of Hematological Disease Models

Hematological diseases originate from hematopoietic stem cells or their differentiated progeny, primarily including BM failure syndromes, myeloproliferative disorders, leukemia, myelodysplastic syndromes, and myelofibrosis. These disorders involve not only genetic or epigenetic abnormalities in hematopoietic cells themselves but are also closely linked to functional alterations in the BM microenvironment [70]. By incorporating patient-derived cells, BM MPS can reconstruct disease-specific microenvironments in vitro, enabling investigation of disease mechanisms and therapeutic responses. Chou et al. [46] constructed a BM chip using CD34^+^ cells from a patient with Shwachman-Diamond syndrome, successfully reproducing the patient’s impaired neutrophil production. They discovered this defect was associated with abnormal proliferation of hematopoietic progenitor cells, demonstrating the utility of BM chips for studying inherited blood disorders.

BM chips have also been employed to study BM fibrosis. Researchers co-cultured MSCs and hematopoietic cells, observing abnormal proliferation of stromal cells and increased cytokine secretion—features consistent with patient BM fibrosis—and demonstrated the chip’s utility for evaluating anti-fibrotic drug effects [71]. Furthermore, the BM MPS can investigate hematopoietic abnormalities associated with infection and inflammation. For instance, adding inflammatory cytokines to the BM chip induces alterations in hematopoietic lineage ratios and immune cell activation, thereby simulating hematopoietic regulation under inflammatory conditions [69].

### 3.5. Tumor Bone Marrow Microenvironment and Bone Metastasis Research

The BM serves as a critical site for bone metastasis in various hematologic malignancies and solid tumors, with the BM microenvironment playing a pivotal role in tumor cell survival, dormancy, and drug resistance. BM MPS, capable of reconstructing interactions between tumor cells and the BM microenvironment, are widely employed in tumor research [72]. Bone metastasis chips constructed on microfluidic platforms provide an experimental framework for deciphering tumor cell bone seeding and early metastasis mechanisms [73]. By integrating vascular endothelial barriers with mineralized bone-like matrix on the chip, these systems enable tracking of cancer cell transendothelial migration, bone matrix adhesion, and cell fate selection within the bone microenvironment, thereby revealing the cellular basis and signaling regulation of bone metastasis [44]. Second, bone metastasis chips have been employed to evaluate drug effects and intervention efficacy across various stages of bone metastasis. For instance, under dynamic perfusion conditions, these chips monitor the regulatory effects of anti-metastatic drugs on cancer cell invasion behavior, bone resorption activity, and tumor signaling pathways, thereby providing an in vitro evaluation platform for screening candidate compounds and therapeutic strategies that effectively inhibit bone metastasis [47].

Regarding application prospects, bone metastasis chips demonstrate potential to develop into personalized risk assessment and drug response prediction platforms. By integrating patient-derived tumor cells with human bone microenvironment components, this system holds promise for achieving differentiated evaluation of drug responses among tumor cells from different patients during the preclinical stage, providing crucial experimental support for developing precision treatment strategies [74].

## 4. Challenges and Perspectives

The in vivo BM is a highly complex, multiscale tissue system. Although existing BMOs and chips can integrate some key cell types, they still struggle to fully reconstruct the marrow’s complete cellular composition and spatial architecture. For instance, most models lack a complete neuromodulatory system, despite evidence that sympathetic nerve signaling participates in HSC mobilization and circadian rhythm regulation [75]. Furthermore, the role of BM adipocytes in regulating hematopoiesis and disease development is gaining increasing attention, yet they remain underrepresented in most MPS [76]. Bone tissue itself possesses a complex mineralized matrix and porous architecture; current engineered bone tissues struggle to fully replicate the nanoscale and microscale features of natural trabecular bone [77].

Currently, BM MPS lack standardized construction protocols. Significant variations exist across studies in cell sources, material compositions, and chip designs. This limits comparability between laboratories and increases batch-to-batch variability in data [78]. Particularly in models relying on primary cells or patient-derived tumor cells, even minor biological differences between donors can potentially affect model functionality. Furthermore, microfluidic chip fabrication involves complex processes including lithography, soft lithography, and material handling, where batch-to-batch variations may compromise experimental outcomes [79]. Biological materials such as collagen or Matrigel inherently exhibit batch variability, thereby affecting microenvironmental stability [80]. Establishing standardized workflow protocols and quality control systems for BM MPS is thus a critical prerequisite for advancing their widespread application in both basic research and industrial settings.

Overall, BM MPS provide crucial human-derived in vitro models for studying hematopoietic regulation and BM diseases. However, challenges remain, including insufficient reconstruction of microenvironmental complexity, limited long-term functional maintenance, inadequate standardization, and clinical translation hurdles. Looking ahead, for BM biomimetic models to achieve regulatory acceptance, simultaneous advancement is needed in both technological refinement and evaluation system development. Technologically, core construction parameters should be defined around key structures and culture conditions, gradually establishing reference materials and performance metrics for comparison to reduce systematic variations across laboratories and platforms. Evaluation-wise, stronger correlations between model outputs and clinically relevant endpoints are needed, ensuring models not only accurately reflect BM biology but also provide predictive value in specific application scenarios. Future integration of advanced biomaterials, 3D bioprinting, multi-organ-on-a-chip technologies, and patient-derived cell sources holds promise for constructing BM models that more closely mimic in vivo physiological states, thereby advancing their application in drug development and precision medicine.

## 5. Conclusions

BM MPS have emerged as promising in vitro platforms for reconstructing the structural and functional characteristics of the hematopoietic niche. By integrating key cellular components, biomaterial scaffolds, vascular structures, and dynamic microfluidic regulation, these systems provide a more physiologically relevant representation of the BM microenvironment than conventional two-dimensional cultures or animal models. Current engineering strategies mainly include self-organized BMOs and microfluidic BM-on-a-chip platforms. Organoid-based systems emphasize multicellular self-organization and three-dimensional microenvironmental reconstruction, whereas microfluidic platforms enable precise control of spatial architecture, perfusion dynamics, and biochemical gradients. Together, these approaches have enabled significant progress in modeling hematopoietic stem cell maintenance, investigating niche regulatory mechanisms, evaluating drug-induced BM toxicity, and studying tumor–BM interactions such as bone metastasis. Nevertheless, important challenges remain, including incomplete reconstruction of the cellular and structural complexity of the BM microenvironment, limited long-term functional stability, and the lack of standardized fabrication and evaluation protocols across laboratories. Future advances in biomaterials, microfluidic engineering, 3D bioprinting, and patient-derived cell technologies are expected to further enhance the physiological relevance and reproducibility of these models. Continued interdisciplinary development will strengthen the role of BM MPS as valuable platforms for hematopoietic research, drug development, and precision medicine.

## Figures and Tables

**Table 1:** Representative biomaterials and their functional roles in BMO construction.

Category	Biomaterial	Key Properties	Function in BM Organoids	Ref.
Natural biomaterials	Collagen (Type I/III)	High biocompatibility; fibrillar ECM structure	Supports 3D scaffold formation and cell adhesion	[31]
	Matrigel	Rich in laminin, collagen IV, growth factors	Promotes cell self-organization and niche formation	[32]
Mineralized biomaterials	Hydroxyapatite (HA)	Bone-like mineral composition; osteoinductive	Promotes osteogenic differentiation and endosteal niche formation	[33]
Synthetic biomaterials	PEG-hydrogel	Tunable stiffness; modifiable chemistry	Enables precise control of stem cell behavior and niche properties	[34]
	GelMA-hydrogel	Highly efficient; tunable structure	Enables 3D assembly of microspheres, promoting self-organization of endochondral ossification	[35]
	Collagen-hydrogel	Viscoelastic; tunable mechanical properties	Provides a viscoelastic 3D matrix that supports organoid integration and formation of continuous tissue structures	[36]
	Hybrid hydrogels	Combines bioactivity and tunability	Mimics ECM while enabling controlled signaling	[37]

**Table 2:** Representative Bone Marrow-on-a-Chip Platforms.

Platform/Model	Key Components	Microenvironment Features	Applications	Ref.
Bone marrow-on-a-chip microfluidic model	Hematopoietic stem/progenitor cells, bone marrow stromal cells	Microfluidic perfusion system mimicking the bone marrow niche	Modeling hematopoietic stem cell maintenance and niche physiology	[28]
Long-term hematopoietic stem cell culture BM-chip	Human hematopoietic stem cells, stromal cells	3D microfluidic culture environment enabling long-term cell maintenance	Studying hematopoiesis and stem cell–niche interactions	[41]
Vascularized human bone marrow-on-a-chip	Endothelial cells, stromal cells, hematopoietic cells	Multi-niche microvascularized bone marrow model with endosteal niche	Investigating bone marrow physiology and niche-specific regulation	[42]
Microfluidic vascularized bone tissue model	Osteoblasts, endothelial cells	Hydroxyapatite-incorporated ECM within a microfluidic device	Studying vascularized bone microenvironment and tissue interactions	[43]
Human bone perivascular niche-on-a-chip	Mesenchymal stromal cells, endothelial cells, tumor cells	Perfusable microvascular niche within an engineered bone microenvironment	Modeling metastatic colonization of cancer cells in bone marrow	[44]
Bone marrow sinusoidal niche microfluidic device	Multiple myeloma cells, endothelial cells	Microfluidic model mimicking bone marrow sinusoidal structures	Studying trafficking of multiple myeloma cells	[45]
Human bone marrow chip for drug toxicity modeling	CD34^+^ hematopoietic progenitors, stromal cells	Two-channel perfusable microfluidic BM-chip	Recapitulating clinical bone marrow toxicities and drug responses	[46]
Bone marrow chip for biologics safety profiling	Hematopoietic and stromal cells	Microfluidic bone marrow model for preclinical drug testing	Safety profiling of biologics in drug development	[47]

## Data Availability

No new data were created or analyzed in this study. Data sharing is not applicable to this article.
